# Integration of Marine Macroalgae (*Chaetomorpha maxima*) with a Moving Bed Bioreactor for Nutrient Removal from Maricultural Wastewater

**DOI:** 10.1155/2020/8848120

**Published:** 2020-06-17

**Authors:** Xian Li, Yale Deng, Xueying Li, Xiaona Ma, Jinxia Wang, Jun Li

**Affiliations:** ^1^Key Laboratory of Experimental Marine Biology, Institute of Oceanology, Chinese Academy of Sciences, Qingdao 266071, China; ^2^Laboratory for Marine Biology and Biotechnology, Qingdao National Laboratory for Marine Science and Technology, Qingdao 266071, China; ^3^Center for Ocean Mega-Science, Chinese Academy of Sciences, Qingdao 266071, China; ^4^Aquaculture and Fisheries Group, Department of Animal Sciences, Wageningen University, Wageningen 6708, Netherlands

## Abstract

Rather than direct nutrient removal from wastewaters, an alternative approach aimed at nutrient recovery from aquacultural wastewaters could enable sustainable management for aquaculture production. This study demonstrated the feasibility of cultivating marine macroalgae (*Chaetomorpha maxima*) with a moving bed bioreactor (MBBR-MA), to remove nitrogen and phosphorus in aquaculture wastewater as well as to produce macroalgae biomass. MBBR-MA significantly increased the simultaneous removal of nitrate and phosphate in comparison with only MBBR, resulting in an average total nitrogen (TN) and total phosphorus (TP) removal efficiency of 42.8 ± 5.5% and 83.7 ± 7.7%, respectively, in MBBR-MA while MBBR had no capacity for TN and TP removal. No chemical oxygen demand (COD) removal was detected in both reactors. Phosphorus could be a limiting factor for nitrogen uptake when N : P ratio increased. The recovered nitrogen and phosphorus resulted in a specific growth rate of 3.86%–10.35%/day for *C. maxima* with an uptake N : P ratio of 6. The presence of macroalgae changed the microbial community in both the biofilter and water by decreasing the relative abundance of Proteobacteria and Nitrospirae and increasing the abundance of Bacteroidetes. These findings indicate that the integration of the macroalgae *C. maxima* with MBBR could represent an effective wastewater treatment option, especially for marine recirculating aquaculture systems.

## 1. Introduction

Nitrogen and phosphorus removal from wastewater in aquaculture production systems is crucial to reduce the eutrophication of receiving water and to ensure the sustainable development of the industry. The limited availability of land and water resources is restricting the further expansion of aquaculture. There has been growing interest in the development of intensive land-based marine aquaculture, especially recirculating aquaculture systems (RASs), in which water can be reused after a series of treatment processes [1]. A biofilter is the core water treatment unit in an RAS, in which ammonia, as the most toxic form of nitrogen, is converted to nitrite by ammonia-oxidizing bacteria (AOB) and then further converted by nitrite-oxidizing bacteria (NOB) to nitrate [2]. A moving bed biological filter (MBBR) is widely used in RASs due to its advantageous properties, including sufficient mixing, effective mass transfer, high removal rate of pollutants, and relatively small spatial requirements [3]. However, the nitrate and phosphorus concentration tends to accumulate to a high value in an MBBR system, and nitrate even could reach hundreds of mg/L [4–6]. It has been shown that high nitrate concentrations can also pose a potential hazard to the cultured species [7–9]. Without proper treatment, the nitrate and phosphorus discharged in the saline wastewater can lead to eutrophication of the adjacent ecosystems [10].

Biological denitrification that converts nitrate and nitrite to nitrogen gas has been applied in RASs [11]; however, this process faces operational challenges, such as the requirement for a large carbon input, technical management, and nitrous oxide emissions [12, 13]. Moreover, eliminating nitrate from the system through nitrogen gas reduces the nutrient recovery efficiency from wastewater. Phosphorus is considered a growth-limiting nutrient in many water systems, which could be removed by plants, microorganism, and chemicals, while no effective approaches have been developed in RAS for phosphorus, especially dissolved phosphorus elimination. Yogev et al. [14] combined denitrification with anaerobic digestion to recover phosphorus by its sustainable reuse as a fertilizer for plants in fresh RAS. Macroalgae have been used for water purification due to their high nitrogen and phosphate removal efficiencies, which make them suitable for biomass production and as a resource for biofuel feedstocks [15, 16]. The use of macroalgae could significantly reduce the harvesting and dewatering costs compared with microalgae due to their larger size and tendency to grow as dense floating mats or substrate-attached turfs [17]. Macroalgae are usually used in integrated multitrophic aquaculture (IMTA) systems to maintain water quality and to serve as food for cultured species [18]. Bambaranda et al. [19] tried to use *Caulerpa lentillifera* as a bioremediatory species instead of MBBR for nutrient removal in a RAS where a huge volume of macroalgae was needed to achieve the required high efficacy. However, no studies have investigated the feasibility of incorporating macroalgae with an MBBR within marine wastewater treatment.

Nambiar and Bokil [20] proposed the concept of a microalgae-bacteria consortia to study the uptake of nitrogen, and several researches were carried to study the interactions of microalgae and bacteria [21]. Both macroalgae and microalgae have numerous effects on microbial community structure and aquatic organisms by directly altering water nutrients, moderating hydrological conductivity, transporting oxygen through their bodies, and secreting chemicals as catalysts [22, 23]. Some bacteria and algae in the system have a mutually beneficial relationship, through which bacteria can convert ammonia into nitrite or nitrate, which can be utilized by algae, while the algae produce oxygen and organic matter for the growth of bacteria. Compared with the traditional single-step treatment, the nitrogen removal efficiency in aquaculture water can be improved by exploiting the synergistic effect that occurs among multiple species [24].

*Chaetomorpha maxima*, which is known for its blooming nature as well as its tolerance to fluctuating aquarium conditions, is a suitable marine macroalgae (MA) species for aquaculture wastewater nutrient recovery and biomass production [25]. And in our preliminary study, *Chaetomorpha maxima* is a good feed candidate for sea cucumber. In this study, integrated MBBR-MA (*Chaetomorpha maxima*) circulating systems were built to evaluate the nitrogen and phosphorus removal performance as well as macroalgae biomass production by comparing them with MBBRs with no macroalgae culture. How the inclusion of macroalgae influenced the microbial community composition in both water bodies and the packed biofilters was also analyzed. This study provides the basis for the application of the MBBR-MA system, which could reduce nutrient accumulations in aquaculture wastewater and improve the sustainability of aquaculture production system.

## 2. Materials and Methods

### 2.1. Experimental Setup and Operational Procedure

**Figure 1:**
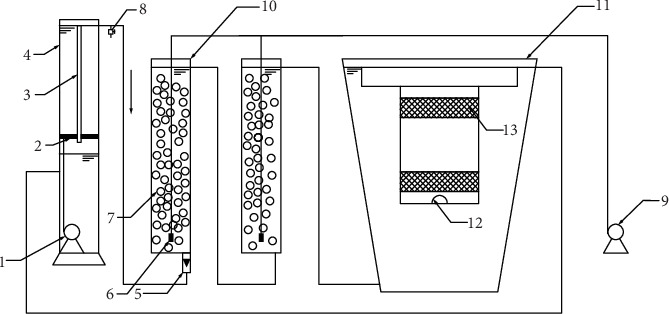
Schematic of the recirculating marine macroalgae with a moving bed bioreactor (MBBR-MA) system. 1, submerged pump; 2, baffle plate; 3, overflow pipe; 4, water storage tank; 5, flowmeter; 6, air stone; 7, biofilm carrier; 8, water sample tap; 9, aeration pump; 10, moving bed biofilm reactor; 11, algae bioreactor; 12, underwater light; 13, sieve.

A schematic of the integrated recirculating MBBR-MA system is shown in Figure 1. The MBBR-MA system included a 20 L water storage tank, two 3.5 L MBBRs, and a 150 L upflow algae reactor. The laboratory-scale MBBR was packed with ring plastic suspension filler (64 holes) to a filling rate of 30%. The MBBR was covered by the blackout cloth to avoid light. The water in the storage tank was lifted by a submerged pump to the MBBR and then to the algae reactor and finally flowed back to the storage tank. An underwater light at 15000 lx light intensity was set in the algae reactor, and the ratio of red to white light was 1 : 3.

Three replicates of two treatments, MBBR-MA and MBBR, were used to test the nutrient removal performance. At the start of the experiment, 50 g of MA (*Chaetomorpha maxima*) was stocked in each of the three algal reactors in the MBBR-MA treatment, while no MA was stocked in the MBBR treatment. The systems were fed with simulated maricultural wastewater prepared using fermented Atlantic salmon residual excrement [26]. The residual excrement was dried at 105°C for 48 h and then broken into a powder and mixed with seawater. A solution of the mixture was anaerobically fermented in a sealed container for 5 d before use. The influent wastewater quality was adjusted as follows: NH_4_^+^-N, 2.0 ± 0.5 mg/L; NO_2_^−^-N, 0.1 ± 0.05 mg/L; NO_3_^−^-N, 2.0 ± 0.5 mg/L; PO_4_^3-^-P, 0.2 ± 0.5 mg/L; total P, 0.3 ± 0.05 mg/L; chemical oxygen demand (COD), 3.00 ± 0.5 mg/L, and Salinity, 31‰. Both systems were operated in recirculating mode at a rate of 7.5 mL/min to achieve a hydraulic retention time (HRT) of 24 h. The experiment lasted for 75 d, consisting of 15 cycles, with each cycle operating for 4 d, with an interval of 1 d to change the wastewater. The whole experimental period was divided into two stages, with stage I lasting for 10 cycles and stage II lasting for 5 cycles. Stage I was designed to evaluate nutrient removal efficiencies between MBBR-MA and MBBR. The fresh algae in the MBBR-MA systems were weighed after each cycle. Stage II was designed to verify whether the MBBR could recover after the removal of the algae reactor. After stage I, the algae in the MBBR-MA systems were removed. All the algae bioreactors were cleaned, and all the underwater lights were removed.

### 2.2. Water and Biofilm Collection

Water samples (250 mL) were taken from the water sample tape from the outlet of the algae bioreactor of all the six systems every 12 h for the analysis of total ammonia nitrogen (TAN), nitrite nitrogen (NO_2_^−^-N), nitrate nitrogen (NO_3_^−^-N), total nitrogen (TN), total phosphorus (TP), and chemical oxygen demand (COD) according to standard methods [27]. All the water samples were stored at 4°C immediately after the collection and were analyzed for 12 hours. Microbial samples in both the biofilter and water were taken at the end of stage I (10 cycles) from the six systems. In detail, 15 plastic suspension fillers were randomly selected from each MBBR system. The biofilms attached to the fillers were collected by shaking in 30 mL of sterile seawater with 100 *μ*L of stabilizer (Tween 80 detergent solution) using a vortex mixer for 10 min. Then, the solution was filtered through a 0.22 *μ*m polycarbonate filter (Millipore, Burlington, MA, USA) to collect the microorganisms. Similarly, 500 mL of water sampled from each system was filtered through a 0.22 *μ*m polycarbonate filter (Millipore, MA, USA) to collect the microorganisms in the water. All the processed samples were stored at −80°C prior to DNA extraction.

### 2.3. DNA Extraction and High-Throughput Sequencing

The total DNA on the filter was extracted with an E.Z.N.A.® Water DNA Kit (Omega Bio-Tek, Norcross, GA, USA) according to the manufacturer's protocol. The DNA from the 12 microbial samples (six biofilm microbial samples and six water microbial samples) were profiled by polymerase chain reaction (PCR) amplification using 515F and 806R primers. The PCR products were separated by electrophoresis on 2% agarose gel, purified using a SanPrep DNA Gel Extraction Kit, and then quantified with NanoDrop. The purified mixtures were finally deep sequenced on the HiSeq2500 sequencing platform (Illumina, San Diego, CA, USA).

### 2.4. Processing of Sequencing Data

Raw data from the HiSeq2500 sequencing platform was processed with Cutadapt and the UCHIME algorithm to obtain clean reads [28, 29]. The clean reads without chimeras were clustered into operational taxonomic units (OTUs) at 97% similarity with UPARSE [30]. Representative sequences processed with QIIME 1.9.1 were used for taxonomic assignments based on the SILVA [31] and SSUrRNA databases [32]. Alpha diversity index values (Chao1 richness estimator, Shannon index, and Simpson index) were obtained using the QIIME 1.9.1 package. A hierarchical cluster heat map was generated, and a principal coordinate analysis (PCoA) was performed based on weighted UniFrac distances of the detected OTUs, with the R package *vegan*.

### 2.5. Statistical Analyses

The specific growth rates (SGR, %/d) of macroalgae were determined using the following formula:

(1) $$\begin{array}{c} \text{SGR}=\left[{\left(\frac{W_{t}}{W_{0}}\right)}^{1/t}-1\right]\times 100\%, \end{array}$$

where *W*_0_ (g) is the initial fresh weight, *W*_*t*_ (g) is the fresh weight at time *t*, and *t* (d) is the time interval.

The nitrogen, phosphorus, and COD removal efficiency (RE) was calculated using the following equation:

(2) $$\begin{array}{c} \text{RE}=\frac{C_{0}-C_{t}}{C_{0}}\times 100\%, \end{array}$$

where *C*_0_ (mg/L) and *C*_*t*_ (mg/L) are the nitrogen, phosphorus, or COD concentrations in the water at the start and after that each cycle, respectively.

Statistical analyses were performed using SPSS 20.0 software and included a Student *t*-test and one-way analysis of variance (ANOVA). Data was reported as the mean ± standard deviation (SD). The data were subjected to a Student *t*-test. A *p* value of <0.05 was considered statistically significant.

## 3. Results and Discussion

### 3.1. Long-Term Nitrogen and Phosphorus Removal Performance

**Figure 2:**
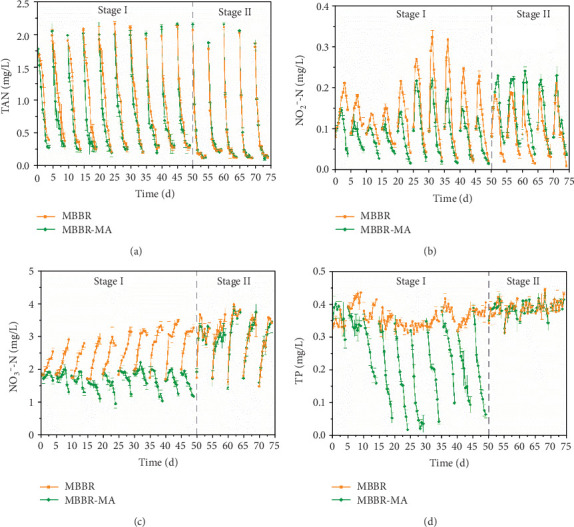
The concentrations of TAN (a), NO_2_-N (b), NO_3_^−^-N (c), and TP (d) dynamics in both MBBA and MBBA-MA.

The variation in the TAN, NO_2_^−^-N, NO_3_^−^-N, and TP dynamics in both the MBBR-MA and MBBR systems is shown in Figure 2. The TAN concentrations at the start of each cycle were 2.02 ± 0.10 mg/L and were reduced to 0.23 ± 0.07 (MBBR-MA) and 0.24 ± 0.08 (MBBR) mg/L at the end of each cycle (Figure 2(a)). In stage I, TAN removal followed zero-order elimination kinetics in the MBBR system during the first four days of a cycle, while the MBBR-MA system had a higher TAN removal rate, indicating that MA contributed to TAN absorption, with the microorganisms in the MBBR playing the main role in TAN transformation. This result was in line with that of stage II, when MA was removed from the algae reactor in the MBBR-MA system, with no difference detected in TAN elimination in both systems.

Nitrite, as an intermediate product of nitrification, accumulated to a peak of 0.22 ± 0.06 mg/L during the first two days and was further oxidized to nitrate in the MBBR system (Figure 2(b)). During stage I, the nitrite accumulation in the MBBR system was significantly (*p* < 0.05) higher than that in the MBBR-MA system, which could be attributed to the nitrite and ammonia assimilation by MA and relatively lower ammonia concentration utilized by bacteria in the MBBR-MA system. Therefore, when the algae were removed from the MBBR-MA in stage II, the sudden increase in the TAN loading caused a slightly higher nitrite accumulation in the MBBR-MA than in the MBBR. The nitrification process resulted in a constant nitrate accumulation of an average of 3.25 ± 0.37 mg/L in the MBBR (Figure 2(c)). On the other hand, the nitrate concentration decreased to 1.00 ± 0.10 mg/L at the end of stage I in the MBBR-MA system (Figure 2(c)), which could be explained by the absorption of MA. Direct nitrate adsorption by MA played a significant role in the nitrate reduction, which was confirmed by Ge and Champagne [17]. When MA was removed from the algae reactor in the MBBR-MA system at stage II, nitrate accumulation reoccurred.

As expected, MBBR had no contribution to phosphorus removal (Figure 2(d)). The biological removal of phosphorus is based on the phosphate-accumulating organisms (PAOs) under anaerobic and aerobic or anoxic conditions through sludge recycling [33]. The aerobic process of MBBR cannot enrich PAOs in the biofilm, which causes phosphorus accumulation in the system. On the hand, TP was eliminated from 0.35 mg/L in the influent to 0.06 mg/L in effluent of the MBBR-MA system after the acclimation of marine macroalgae for three cycles. The high capacity of TP removal in MBBR-MA was attributed to the uptake of phosphorus compounds for photosynthesis of marine macroalgae [34]. In our study, the average initial influent N : P ratio in MBBR-MA was 12.4 while the average uptake N : P ratio of MA was 6.0 at day 4 during cycle 4 to cycle 10 in stage I. A previous study showed that N : P ratios greater than 15 in a temperate region indicated phosphate limitation for several species of MA [35]. The higher N : P ratio in wastewater influent than that in macroalgae uptake implied that phosphorus availability was a limiting factor for C. *maxima* growth, inhibiting the further uptake of nitrogen from aquaculture wastewater.

### 3.2. Nutrient Removal Efficiency within a Cycle

**Figure 3:**
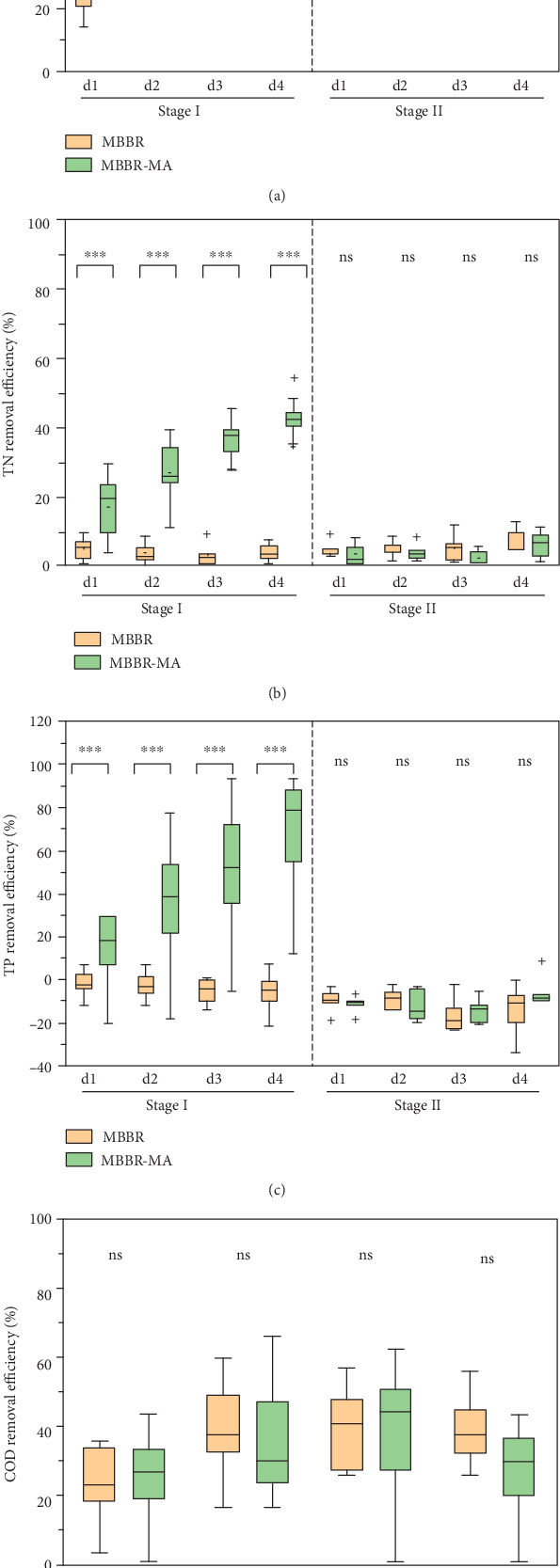
The TAN (a), TN (b), TP (c), and COD (d) removal efficiency over the four days in stage I and stage II of both MBBR and MBBR-MA.

The nutrient removal efficiency over the four days within a cycle is shown in Figure 3. The TAN removal efficiency increased gradually from day one to day four in each cycle (Figure 3(a)). In stage I, the average accumulating TAN removal efficiency was 54.9% (MBBR-MA) and 33.2% (MBBR) on the first day, 70.8% (MBBR-MA) and 53.8% (MBBR) on the second day, 84.1% (MBBR-MA) and 77.4% (MBBR) on the third day, and 86.9% (MBBR-MA) and 86.0% (MBBR) on the fourth day. It was reported by the fact that *C. maxima* prefers to absorb ammonia as nitrogen source than nitrate when ammonia concentration is higher than 1.5 mg/L and prefers to absorb nitrite and nitrate when ammonia dropped below 1.5 mg/L [25]. Those results indicated that MA only contributed to TAN removal in MBBR-MA mainly on the first day when the TAN concentration was higher than 1.0 mg/L (Figure 2(a)). However, the difference between the two groups became insignificant on d4, indicating that microorganisms attached to MBBR played a significant role in TAN transformation. This assumption can be verified in stage II that there was no significant (*p* < 0.05) difference between the two treatments in TAN removal efficiency when macroalgae was removed.

In an MBBR, nitrification is a major ammonia transformation process, by which ammonia is oxidized to nitrite and further oxidized to nitrate. In stage I, the accumulation of NO_3_^−^-N was consistent with the degradation of TAN caused by the nitrification process in the MBBR which explained the zero removal of TN in MBBR (Figure 3(b)). The decrease of nitrate mainly started from d2 in MBBR-MA (Figure 2(c)), implying that *C. maxima* first utilize ammonia as nitrogen source then switched to nitrate when ammonia was lower than approximate 1.0 mg/L. Overall, the direct absorption of nitrogen by *C. maxima* contributed to the TN removal in the MBBR-MA, with an average removal efficiency of 42.8% for TN, ranging from 34.4% to 54.3% at day 4 (Figure 3(b)). *C. maxima* exhibited higher removal efficiency in TP removal (average, 80.0%) when compared with TN removal (Figure 3(c)). This indicated that the relatively low phosphorus availability in aquaculture wastewater limited the nitrogen uptake by MA. Nevertheless, since autotrophic macroalgae uptake no carbon from the wastewater (Figure 3(d)), the lower nitrate level in the MBBR-MA system may elevate the C : N ratio that could facilitate the denitrification process, which could benefit the zero-exchange operation of RAS.

### 3.3. Characteristics of MA Growth

The algal growth was described as the weight gain and SGR of MA on a fresh weight basis (Table 1). The fresh weight increased and the rate of growth gradually accelerated over the course of the study. At the end of the tenth period, the fresh weight of MA was 564.03 g, i.e., 11 times the original algae weight. These results confirmed that MA can use inorganic nitrogen for their growth, contributing to aquaculture water purification [36–38]. However, the growth performance of the macroalgae varied according to the species used for cultivation. For example, *Ulva lactuca* grown in a mixture of seawater and sewage effluent (40%:60%) had an SGR of 0.5%/day [37]. In Brazil, a study of the growth and biofiltration capacity of the macroalgae *Gracilaria birdiae* in tank cultivation had an SGR of 3.6%/day [38]. Our study revealed a significant growth performance, with the SGR varying from 3.86 to 10.35%/day, indicating that the application of *C. maxima* could be an optimal algae alternative when treating maricultural wastewater.

### 3.4. Characteristics of Microbial Diversity in Water and Biofilter

#### 3.4.1. Alpha-Diversity Analysis

A total of 855,846 high-quality 16S rRNA gene sequence reads were obtained from the 12 biofilter and water samples. Each library contained 56,298–87,910 reads that were normalized to 56,298 reads for the comparison of microbial community diversity between the MBBR-MA and MBBR systems (Table 2). Water samples had a significantly (*p* < 0.05) higher microbial richness than biofilter samples according to the OTU number and Chao1 index values, while the addition of macroalgae did not significantly (*p* < 0.05) change the microbial richness in biofilters. Moreover, the Shannon and Simpson indices indicated that MA did not significantly change the evenness of the microbial community in biofilter samples. The Simpson index was significantly higher in the water samples with algae than in those without algae. The results of the alpha diversity analysis showed that the microbial community richness and diversity were lower in the biofilm than in the water samples, particularly for the Shannon and Simpson index results (*p* < 0 05), which was in line with previous studies [39, 40].

#### 3.4.2. Beta Diversity Analysis

**Figure 4:**
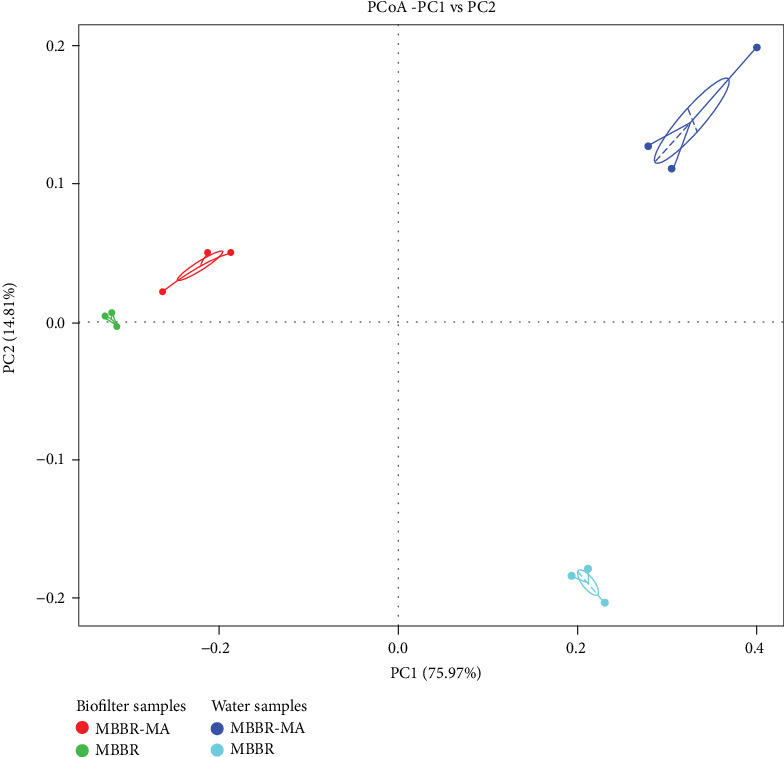
The microbial community distribution in both biofilter and water samples according to a principal coordinate analysis (PCoA) plot of MBBR and MBBR-MA.

The difference in microbial composition between systems was analyzed by the weighted UniFrac distance and presented as a PCoA plot (Figure 4). A clear separation between the biofilter and water samples was observed along PC1 axes, which accounted for 75.97% of the total changes in the bacterial community composition. Many aquatic microorganisms are capable of colonizing surfaces, leading to the formation of biofilms with specialized properties [39, 40]. Thus, in terms of diversity (Table 2) and composition, the bacterial community in the water and biofilters was clearly different, which reflected the different microbiota compositions attached to RAS biofilters compared to the free-living community in the water phase of aquaculture systems [41].

The samples could be separated between MBBR-MA and MBBR along PC2, which explained 14.81% of the total variation in the bacterial community composition. This result demonstrated that the microbial communities in both the biofilm and water samples were influenced by the presence of macroalgae. The MA induced a bigger shift in the microbial community in the water than in the biofilters. One reason for this was that the utilization of CO_2_ and release of oxygen during photosynthesis might change the pH in the water and further influence the microbial community composition [42, 43]. Another possible reason could be that that the reduction in the nitrogen concentration by algae could also affect the microbial communities in MBBR, as suggested by previous studies [7, 44].

### 3.5. Microbial Community Composition

**Figure 5:**
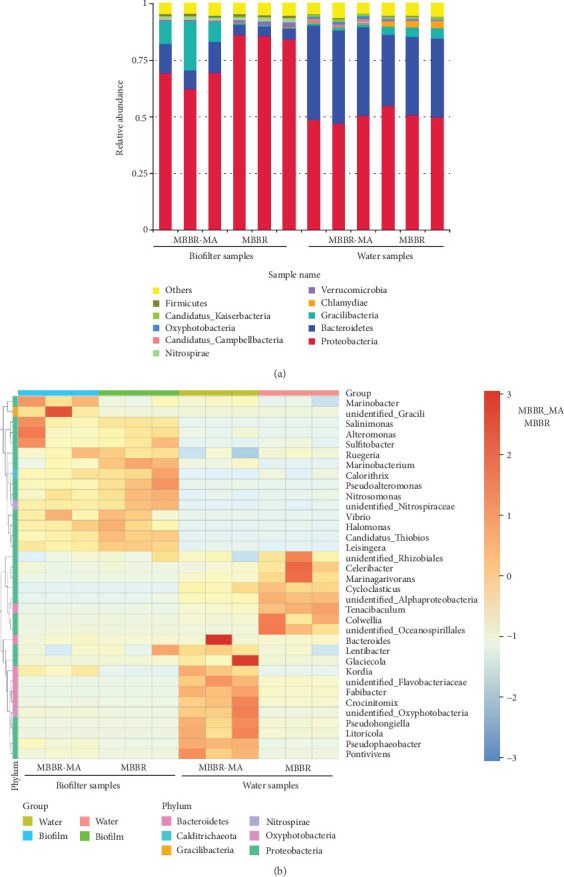
The microbial community composition of biofilm and water samples of MBBR and MBBR-MA at the (a) phylum taxon level and (b) genus taxon level.

The microbial community composition of the biofilm and water samples was analyzed at phylum, class, and genus taxon levels (Figure 5). At the phylum level, the top 10 phyla were selected, and the remaining phyla were assigned to a cluster named “the others” (Figure 5(a)). The results showed that Proteobacteria (average relative abundance (RA), 63.4%) was the most abundant phylum across all the samples, followed by Bacteroidetes (average RA, 22.4%), which was also found with a high abundance in other marine MBBRs [45–47]. The integration of MA with MBBR significantly decreased the RA of Proteobacteria and Nitrospirae and significantly increased the RA of Bacteroidetes in both biofilter and water samples. Nitrospirae is the dominant NOB in wastewater treatment systems [48], and many Bacteroidetes species have been reported to have the capacity for organic carbon degradation [49]. The relatively lower abundance of Nitrospirae in MBBR-MA biofilter samples could be explained by the relatively lower nitrite concentration detected in the MBBR-MA system (Figure 2(b)), because macroalgae also contribute to ammonia and nitrite removal. On the other hand, macroalgae may also enhance the growth of heterotrophic bacteria by producing organic compounds.

A heat map of the 35 most abundant genus-level taxa is shown in Figure 5(b). A clear separation between biofilter and water samples was observed. In the biofilter samples, *Halomonas*, *Leisingera*, *Pseudoalteromonas*, *unidentified_Gracilibacteria*, *Alteromonas*, *Vibrio*, and *Kordia* were the most abundant genera in both treatments. Among them, macroalgae significantly (*p* < 0.05) increased the RA of unidentified *Gracilibacteria* and *Kordia* in the MBBR-MA compared with the MBBR. According to Chen et al. [50], genera belonging to *Gracilibacteria* can participate in the denitrification process and have nitrate removal abilities. According to Paul and Pohnert [51], *Kordia*, as algicidal bacterium, can release protease as an algicidal protein when they receive signals from algae indicating cellular senescence. On the other hand, macroalgae significantly reduced the RA of *Pseudoalteromonas* and *Alteromonas* in the MBBR-MA system compared to the MBBR system. It has been documented that members of the genus *Pseudoalteromonas* are involved in the formation of biofilms [52]. Bacteria belonging to the genera *Pseudoalteromonas* and *Alteromonas* produce depolymerizing enzymes and are associated with macroalgal degradation processes or algicidal activities [53, 54]. The lower abundance of those genera may be beneficial for the growth of macroalgae. Moreover, macroalgae reduced the RA of nitrifying bacteria, including *Nitrosomonas* (0.49% for MBBR-MA, 0.80% for MBBR) and *Nitrospiraceae* (1.22% for MBBR-MA, 1.76% for MBBR), in biofilters. The numerical dominance of NOB (i.e., *Nitrospiraceae*) over AOB (i.e., *Nitrosomonas*) might be a general characteristic of ammonium limited systems [55], which was in agreement with other studies in which the ammonia concentration was relatively low (around 2 mg/L). Most of the dominant microorganisms in the biofilm were present at very low levels in the water samples, which reinforced the above findings of differences in microbiota between water and biofilms.

Intensive interactions were identified between bacteria and macroalgae, including stimulatory and inhibitory effects on each other by modifying the chemical microenvironment of the other group through metabolic activities [55, 56]. Our study revealed that an MA culture integrated with an MBBR changed the microbial community in biofilters, decreasing the abundance of nitrifying bacteria and increasing the abundance of heterotrophic bacteria through the absorption of inorganic nitrogen and release of organic matter. Further research is required to determine how the integration of macroalgae influences the microbial functionality and nitrogen removal efficiency of an MBBR biofilter and to investigate the potential interactions between macroalgae and bacteria.

## 4. Summary and Conclusions

An integrated MBBR-MA circulating system was performed for both nutrient removal and macroalgae biomass production. The MA improved the TN removal efficiency of MBBR from 3.9% to 42.8%, mainly by nitrate absorption, and contributed to 66.8% TP removal which enabled the sustainable operation of a marine RAS. *Chaetomorpha maxima* achieved a specific growth rate of 3.86–10.35%/day through nutrient recovery, and the uptake N : P ratio by MA was 6. Phosphorus could be a limiting factor for *Chaetomorpha maxima* to uptake nitrogen when the influent N : P ratio increased. The high-throughput sequencing results revealed a shift in the microbial composition of both water and biofilter samples in the systems with and without macroalgae.

## Figures and Tables

**Table 1:** The growth performance of marine macroalgae (MA) in each cycle in stage I.

Cycle	0	1	2	3	4	5	6	7	8	9	10
Fresh weight (g)	50.86 ± 0.31	75.42 ± 13.17	94.68 ± 8.88	113.90 ± 11.07	143.54 ± 6.18	182.44 ± 15.46	212.31 ± 7.11	278.28 ± 9.79	329.72 ± 8.02	424.38 ± 15.16	564.03 ± 30.35
Fresh weight gain (g)	—	24.56 ± 9.02	19.26 ± 5.73	19.22 ± 5.76	29.64 ± 6.95	38.90 ± 8.57	29.87 ± 7.03	65.97 ± 10.29	51.44 ± 6.09	94.66 ± 11.33	139.65 ± 14.94
SGR (%/d)	—	10.35	5.85	4.73	5.95	6.19	3.86	7.05	4.28	6.51	7.37

**Table 2:** Alpha-diversity indices of the biofilter and water samples from both MBBR and MBBR-MA.

Sample name (*n* = 3)		ACE	Chao1	Shannon	Simpson	PD whole tree
Biofilter	MBBR-MA	900^a^	901^a^	6.10^a^	0.96^a^	76.73^a^
	MBBR	844^a^	840^a^	6.25^a^	0.97^a^	77.63^a^

Water	MBBR-MA	1094^b^	1096^b^	6.67^b^	0.96^a^	101.84^b^
	MBBR	1194^b^	1183^b^	6.70^b^	0.94^b^	112.17^b^

## Data Availability

The data used to support the findings of this study are included within the article.
